# Cannabis-Based Medicinal Products in the Management of Emotionally Unstable Personality Disorder (EUPD): A Narrative Review and Case Series

**DOI:** 10.3390/brainsci12111467

**Published:** 2022-10-29

**Authors:** Waseem Sultan, Anup Mathew, Matthew R. D. Brown, Juan F. Gálvez-Flórez, Guillermo Moreno-Sanz

**Affiliations:** 1 Surrey and Borders Partnership NHS Foundation Trust, Leatherhead KT22 7AD, UK; 2 Zerenia Clinics, London SW1X 9AE, UK; 3 The Royal Marsden Hospital, London SW3 6JJ, UK; 4 Clinica Zerenia, Bogotá 110221, Colombia; 5 Khiron Life Sciences, 28001 Madrid, Spain

**Keywords:** cannabis, CBMP, EUPD, personality disorder, endocannabinoid system

## Abstract

Emotionally unstable personality disorder (EUPD) is a common mental health disorder, manifesting with a range of chronic and debilitating symptoms, including impaired social functioning, unstable mood, and risky impulsive or self-injurious behaviour. Whilst the exact aetiology has not been fully elucidated, implicated factors seem to include genetic factors, environmental causes such as trauma, and neurotransmitter deficits. The literature suggests that impaired functioning of the endocannabinoid system in key brain regions responsible for emotional processing and stress response may underlie the manifestation of EUPD symptoms. The National Institute for Health and Care Excellence (NICE) 2009 guidelines state that “no drugs have established efficacy in treating or managing EUPD”, and yet, patients are commonly prescribed medication which includes antipsychotics, antidepressants, and mood stabilisers. Here we present a case series of seven participants diagnosed with EUPD and treated with cannabis-based medicinal products (CBMPs). Participants were given an initial assessment and followed up one month after CBMPs prescription. Improvement in symptoms was assessed by the completion of ratified rating scales by the participant and psychiatrist. Our results indicate that CBMPs were effective and well tolerated, as six participants reported a noticeable improvement in their symptoms and functioning. Although promising, further research is needed to ascertain the long-term tolerability, efficacy, and dosing strategy for CBMPs in EUPD.

## 1. Introduction

### 1.1. EUPD

Emotionally unstable personality disorder (EUPD), also termed borderline personality disorder, is a commonly encountered form of personality disorder with an estimated prevalence in secondary care mental health services of 20% [1]. EUPD is a chronic and enduring mental illness, often manifesting with a range of debilitating symptoms that may include significantly impaired personality functioning, feelings of emptiness, unstable mood and relationships, along with dangerous impulsive behaviour, including episodes of self-harm [2,3].

Although the etiopathogenesis and exact nature of EUPD has still not been fully elucidated, research has confirmed a hereditary component [4,5,6,7]. This predisposition can lead to a person becoming more sensitive to environmental cues and, consequently, developing exaggerated negative perceptions of others and a worsening of their mental state. Environmental factors may also be implicated, as a high proportion of patients have reported adversities in childhood, with numerous cases identifying significant traumatic events, such as neglect and abuse [8]. Several theories have suggested neurotransmitter abnormalities, particularly in the serotonergic, dopaminergic and norepinephrine pathways [9,10], however, conclusive evidence for this is still limited [11]. Functional neuroimaging studies have documented how core features of EUPD are related to impairments in distinct neural circuits regulating the control of emotional processing, such as the amygdala–prefrontal cortex (PFC) circuit [12].

Despite the frequency of its occurrence and the potential for detrimental effects on patients’ mental wellbeing and functional levels, the therapeutic options available to clinicians managing EUPD are limited. Whilst therapies such as dialectical behavioural therapy and mentalisation therapy have been shown to be efficacious in reducing the severity of symptoms like suicidality, therapy is not always easily accessible, can have long waiting lists, and requires the patient to be engaged in the process which can be extremely difficult when going through an acute crisis [13]. The UK National Institute for Health and Care Excellence (NICE) 2009 guidelines state that ‘no drugs have established efficacy in treating or managing borderline or antisocial personality disorder’. In addition, ‘antipsychotic and sedative medication can sometimes be helpful in the short-term management of crisis (the duration of treatment should be no longer than 1 week for sedative medication) or treatment of comorbid conditions’ [14]. However, despite this advice, a significant proportion of patients with a diagnosis of EUPD, some reports suggesting up to 90%, are prescribed at least one psychotropic medication including antipsychotics, antidepressants, mood stabilisers, and sedatives [15], with polypharmacy being common [16]. Most of these medications are commonly associated with side effects and are prescribed off-label for this indication [17,18,19]. Whilst there is an indication that certain medications can help with specific symptoms, the overall prognosis of the condition is unaltered by their use [15]. It is in this context that we explore and discuss the role of the endocannabinoid system (ECS), a neurochemical system that may provide a novel treatment pathway for the symptoms of EUPD. 

### 1.2. The Neuromodulating Effects of Cannabinoids

Cannabis contains approximately 400 distinct chemical compounds (phytochemicals), including over 100 cannabinoids, the most prominent being Δ9-tetrahydrocannabinol (THC) and cannabidiol (CBD) [20]. THC exerts its pharmacological activity mainly by engaging with type-1 and type-2 cannabinoid receptors, namely CB1 and CB2 receptors. These are G protein-coupled receptors that are expressed in numerous cell types throughout the body but differ significantly in their tissue distribution. CB1 receptors are present in large quantities in the central and peripheral nervous system, gastrointestinal tract, skeletal muscle, and adipose tissue, whilst CB2 receptors are mainly found on immune cells, including the glia, where they modulate cytokine release amongst other functions [21]. Cannabinoid receptors are activated by the endogenous cannabinoids (endocannabinoids) arachidonoylethanolamide (anandamide, AEA). And 2-arachidonoylglycerol (2-AG). These are bioactive substances derived from arachidonic acid and produced on demand from membrane phospholipids [22]. 

**Figure 1:**
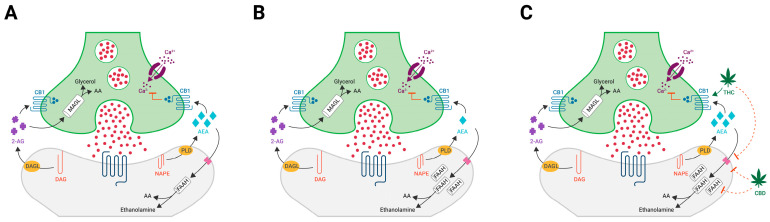
A series of diagrams illustrating the production of endogenous cannabinoids 2-AG and AEA from precursor molecules, their mechanism of action at the presynaptic CB1 receptors (with the resultant effect of reduced presynaptic calcium influx and hence neuronal excitement), and subsequent degradation by FAAH and MAGL. (**A**) Normal ECS functioning; (**B**) the ECS in EUPD showing an increase in FAAH-mediated degradation of AEA; (**C**) potential exogenous cannabinoid mechanism of action at the presynaptic CB1 receptor and at the postsynaptic deactivation of anandamide.

Cannabinoid receptors, endocannabinoids, and the enzymes involved in their synthesis and degradation comprise the endocannabinoid system (ECS), a functionally diverse regulator of many physiological processes which favours homeostasis [23]. AEA and 2-AG are synthesised in the postsynaptic neuron, released into the synaptic cleft, and bind as agonists to the presynaptic CB1 receptor located on the axon terminal or the CB2 receptors in glial cells. Via its G-protein coupled mechanism, binding at the CB1 receptor has a resultant effect of attenuating axonal calcium influx [24]. AEA and 2-AG are eventually degraded by hydrolytic enzymes, namely fatty acid amide hydrolase (FAAH) and monoacylglycerol lipase (MAGL) (see Figure 1A). FAAH is found distributed widely in the CNS and cortex, which is also known to have a high preponderance of CB1 receptors. These enzymes function to regulate and control endocannabinoid signalling by modulating the lifespan of endocannabinoid molecules. From a neurobiological perspective, activation of the CB1 receptor has a multitude of additional effects in the central and peripheral nervous system, the net effect of which is to dampen neuronal excitability and modulate neurotransmission. Furthermore, there is substantial preclinical evidence of a range of other impacts at a cellular level, which include CB1 receptor-induced inhibition of new synapse formation and the retraction of neurites resulting in neuronal morphological changes [25] and the modulation of microglial cytokine production in murine models of neuroinflammation [26].

### 1.3. The Endocannabinoid System as a Therapeutic Target for EUPD

It has been postulated that the ECS plays a significant role in the pathogenesis of many psychiatric conditions. The regions of the brain often implicated in many mental disorders, namely the limbic cortex, prefrontal cortex, amygdala, and hippocampus also contain a high concentration of the ECS. This is a dynamic arrangement that may be influenced by external factors; for example, exposure to chronic stress in mice results in significantly increased CB1 receptor agonist binding site density in the prefrontal cortex and a decrease in CB1 receptor agonist binding site density in the hippocampus, hypothalamus, and ventral striatum [24]. Hippocampal dysfunction is associated with an increase in circulating glucocorticoids after exposure to chronic stress, which is thought to occur through negative inhibition of the hypothalamic–pituitary axis (HPA). Due to the high density of CB1 cannabinoid receptors in this region, endocannabinoids may have an important role in this negative feedback loop. Several studies have shown that increased cannabinoid hippocampal signaling can prevent stress-induced behavioural changes [27]. Chronic stress is also associated with relevant physiological changes affecting ECS functioning, both within this circuit but also throughout the CNS. Notable findings include reduced AEA, increased 2-AG, loss of CB1 receptors, and increased FAAH levels. The ECS in the prefrontal cortex, amygdala, and hippocampus is responsible for suppressing the activity of the HPA following acute exposure to stress and thereby modulates the stress response through the reduction of circulating glucocorticoids. The consequences, therefore, of a dysfunctional ECS system are a prolongation of the stress response due to reduced negative feedback from the CB1 receptor system [27]. 

One of the leading hypotheses for the pathogenesis of EUPD is an abnormal function of the amygdala–PFC circuit. This circuit is implicated in higher-order decision-making and appropriate fear response, both of which are typically impaired in patients diagnosed with EUPD. Dysfunction in the PFC may manifest clinically as impaired decision-making or impulsivity and has been noted to be hypo-responsive in functional imaging studies, whereas heightened amygdala response is thought to lead to symptoms of increased negative emotion. The current model, elucidated experimentally by ^11^C-CURB PET functional imaging, suggests that stress and genetic predisposition may increase the local expression of the endocannabinoid-degrading enzyme FAAH in key regions of this circuit, resulting in lower levels of circulating anandamide and leading to emotional dysregulation [12] (Figure 1B). Pharmacological intervention with cannabinoids may help alleviate symptoms of many psychiatric conditions either by directly activating cannabinoid receptors, as is the case with THC, or by restoring endocannabinoid signaling in this crucial circuit. Sustained administration of oral CBD has also been shown to increase circulating levels of anandamide in psychotic patients, which may be achieved by several mechanisms [28] (Figure 1C).

An alternative plausible mechanism for the therapeutical action of cannabinoids in EUPD could be through the control of neuroinflammation, which is increasingly thought to play a key role in the aetiology of several psychiatric disorders including depression, epilepsy, obsessive-compulsive disorder, and schizophrenia [29]. It has become increasingly recognised as a feature of EUPD and is emerging as an area of significant research interest. MacDowell et al. demonstrated that patients with EUPD present increased activation of inflammatory pathways and inhibition of the antioxidant pathway with a partial correlation to impulsivity scores [30]. Exogenous cannabinoids have been studied extensively as emerging anti-inflammatory agents, which are proposed to act as immunomodulators via several mechanisms that may or not be mediated by cannabinoid receptors. First, it is well known that both THC and CBD can induce apoptosis in immune cells with significant levels of THC-induced apoptosis observed in T cells, B cells, and macrophages [31]. Cannabinoids can also impart a protective effect from apoptosis in healthy CNS cells with a possible role in autoimmune conditions such as multiple sclerosis [32]. Preclinical studies have demonstrated that 3D mice brain aggregate cell cultures with CB1 receptor knockout showed greater caspase-3 activations and decreased neurofilament-H expression when exposed to IFN-ϒ compared to wild-type cultures, indicating the neuroprotective role elicited through CB1 receptor activation [33]. In addition to this, cannabinoids are also thought to act via the downregulation of cytokine and chemokine production [31]. This situation is further illustrated by evidence that murine astrocytes exposed to bacterial lipopolysaccharide released significantly less nitric oxide when given AEA compared to a CB1 receptor cannabinoid antagonist [34]. 

There is now acceptance that cannabis has demonstrable beneficial therapeutic effects in conditions including chemotherapy-induced nausea and vomiting, chronic pain, multiple sclerosis, anxiety, sleep disorders, and epilepsy, all within a satisfactory safety profile [35]. Here we report on the use of cannabinoids as a strategy for the clinical symptomatic management of EUPD and associated conditions in a case series of 7 patients in whom such a therapeutic approach has been implemented. Additionally, we explore and discuss the plausible mechanisms of action for this medication at a cellular and molecular level and review the current evidence for the use of CBMP as a novel adjunct treatment for EUPD. 

## 2. Materials and Methods

### 2.1. Participants

We selected a convenience patient population from Zerenia clinics (United Kingdom and Colombia) receiving treatment with CBMPs. Inclusion criteria included patients: (i) with an established diagnosis of EUPD that had trialled at least one medication and one form of psychotherapy, such as cognitive behavioural therapy (CBT) or dialectical behavioural therapy (DBT); and (ii) who had at least one follow-up review at Zerenia clinic with records of patient-reported outcome measures (PROMS). Patients with a diagnosis of “EUPD traits” were excluded, as this does not constitute a formal personality disorder diagnosis. Participants were asked to provide information about their diagnosis, previous treatment, comorbidities, and previous substance/cannabis use. 

### 2.2. Cannabis-Based Medicinal Products

Participants were prescribed CBMPs based on clinical judgement, although consideration was also given to patient preference and individual choice regarding, for example, the route of administration or desired clinical effects. CBMPs available to prescribers at Zerenia clinic UK consisted of dried cannabis flowers (flos) for inhalation and oral extracts of varying THC and CBD relative concentrations. CBMPs available to prescribers at Zerenia Colombia were limited to oral extracts, as dried cannabis flowers are not authorised for medicinal use in the country. Composition of CBMPs was determined by the chemotype: Chemotype 1 (THC >>> CBD), Chemotype 2 (THC ≈ CBD in a 1:1 ratio), Chemotype 3 (THC <<< CBD). The dosing of CBMPs needs to be personalised by slow titration. A protocol for the titration of cannabis flos inhalation was suggested to patients at the initial appointment [36]. The titration of oral CBMPs typically involves increasing the dose every 3–5 days until the patients achieve acceptable symptomatic control or side effects appear [37].

### 2.3. Outcome Measures

All assessments were conducted by the clinic’s consultant psychiatrist and results were further ratified by the completion of validated scoring instruments to measure improvement and change of symptom severity from baseline at the one-month follow-up review with the consultant psychiatrist. As part of the standard clinical procedure, prescribing psychiatrists are asked to rate patient’s improvement by completing the Clinical Global Impression Improvement scale (CGI-I), a 1-item score in a scale of 1 to 7, where 1 represents “very much improved” and 7 represents “very much worse”. Conversely, patients are asked to complete the Patients’ Global Impression of Change scale (PGIC), a 1-item score to describe the change, if any, in activity limitations, symptoms, emotions and overall quality of life related to their condition. Scoring in the PGIC scale ranges from 1 to 7 with 1 being “no change or condition has got worse” and 7 representing “A great deal better, and a considerable improvement that has made all the difference”. Participants are also asked to report any side effects they experienced at each follow-up.

## 3. Results and Discussion 

A total of 7 patient cases from two international centres (the United Kingdom and Colombia) were collated and presented. The basic demographics and clinical presentation are shown in Table 1. As might be expected for patients seeking a prescription of CBMPs, most participants were young, with 5 out of 7 being under the age of 30. This could be due to more awareness of CBMPs or having previously tried illicitly sourced cannabis and benefiting from it. Three participants were unemployed at the time, and whilst the individual reasons are not known, it is common for patients with EUPD to struggle to hold down long-term employment due to disruption in their social functioning and impulsivity. Additionally, five participants presented psychiatric comorbidities, with the most diagnosed conditions being depressive and anxiety disorders. Although this represents a challenge to identify exactly which aspects of the presentation have improved, EUPD patients seen in routine clinical practice often present other comorbid conditions, especially depression, due to the nature of their distress, poor social functioning, and potentially related mechanisms. 

As per UK legislation, all participants had trialled at least one form of talking therapy and at least one medication. A wide range of drugs have been employed, both past and present, including antidepressants, anxiolytics, and antipsychotics (see Table 2). Six participants had tried three or more medications, sometimes in combination, demonstrating the difficulty of managing EUPD in the clinical setting. As expected, five participants had previously tried illicit cannabis prior to presenting at the clinic and only two were cannabis naïve. It has been suggested that illicit cannabis may indeed confer patients some relief in symptoms such as intrusions, flashbacks, irritability, and anxiety [38]. Other studies have reported concerns with regard to an increase in cognitive dysfunction, reduced functioning, and a higher prevalence of depression and suicidal risk with long-term, non-medical cannabis consumption in patients suffering from mood disorders [39]. However, the use of illicit cannabis is often hindered by inherent limitations such as the quality, safety, and availability of the product, making it difficult to achieve consistency. Additionally, the use of illicitly acquired cannabis presents the risk of criminalisation. Therefore, it is not surprising that patients who have previously trialled illicit cannabis and have experienced a therapeutical benefit would seek the means to legally access CBMPs.

Participants were prescribed a form of CBMP as per their symptoms and preference, as outlined in Table 3. The many combinations of dried flowers for inhalation and oral extracts that can be used to suit each patient´s needs make prescribing CBMPs unique. This approach may, on its own, empower the patient to feel a greater sense of control and responsibility, where the very act of prescribing promotes and encourages a collaborative and holistic approach to mental health treatment. Treatments consisted of the following: four patients exclusively received chemotype 1 cannabis flowers (#1, #3, #4, and #6); one patient received a combination of chemotype 1 cannabis flowers and a chemotype 2 oral extract (#2); one patient received a chemotype 2 oral extract (#5); one patient received a chemotype 3 cannabis oral extract (#7). Doses for flowers ranged between 20 g and 60 g per month (0.6–2 g per day). Maximal doses for oral extracts after the titration period were 9–10 mg of THC per day for chemotype 2 extracts and 40 mg/day or CBD for chemotype 3 extracts. Patients #1 to #6 received treatment for approximately 30 days and reported PGIC values at the 1-month follow-up. Patient #7 received treatment for 90 days and PGIC values were collected at the 3-month follow-up. As depicted in Table 3, scoring from the CGI-I and PGIC correlated for all participants, with both the patient and the prescribing psychiatrist in agreement on the degree of overall improvement in the condition. Six participants reported an improvement in symptoms: 5 participants rated their change as a 6 on the PGIC (“*better, and a definite improvement that has made a real and worthwhile difference”*). Alternatively, a more prolonged treatment with higher oral doses of CBD yielded similar outcomes. Intriguingly, the only patient that did not report any benefit (participant #5) was treated with a chemotype 2 oral extract alone, which seems in agreement with the hypothesis that either fast-acting administration of inhaled THC or prolonged accumulation of oral CBD in higher doses may be a more effective strategy to help EUPD patients cope with their symptoms. This is based upon recent clinical studies which demonstrated an association between elevated FAAH activity in key brain regions, such as the prefrontal cortex, and impulsivity/neuroticism seen in EUPD and other personality disorders, such as personality disorder by using functional imaging techniques, such as ^11^C-CURB positron emission tomography (PET) [40]. Circulating levels of AEA appear to be reduced in EUPD patients, potentially due to the observed exacerbated FAAH expression in key neural circuits, which may, in turn, result in a deficient local endocannabinoid tone in key neural circuits (Figure 1B). According to this hypothesis, there could be several plausible mechanisms by which exogenous cannabinoids, such as THC and CBD, might help to reinstate the tonic activation of the ECS at the CNS, thus ameliorating the symptoms seen in EUPD and other related disorders (Figure 1C). Besides direct activation of presynaptic CB1 receptors by THC, both molecules have been shown to bind to fatty-acid binding proteins (FABPs), which may, in turn, slow down the deactivation of anandamide by interfering with its internalisation and degradation by FAAH [41]. Furthermore, repeated administration of oral CBD in high doses (800 mg/day) has been shown to increase circulating levels of anandamide in psychotic patients. It remains unclear, however, if this effect is a consequence of the direct action of CBD on anandamide transport or degradation, or if it is due to the engagement of other molecular targets [42]. Moreover, this improvement in symptoms was felt after just one month of treatment, particularly in patients treated with chemotype 1 cannabis flowers. This differs somewhat when compared to commonly used psychiatric drugs, especially antidepressants, which can take 2–3 weeks for the initial effect but a much longer time for patients to achieve remission [43]. This quick onset may be of value in the so-called “EUPD crisis”, whereby patients who present to mental health services extremely distressed are sometimes prescribed benzodiazepines and other anxiolytic or hypnotic medication that can quickly produce tolerance and dependence, having disastrous long-term consequences.

The treatment was well-tolerated and none of the participants reported any adverse side effects, which is relevant for medication adherence since a qualitative analysis of 36 articles found that medication side effects were accountable for 28% of patient non-adherence to antipsychotics [44]. The same study reported that a negative attitude towards medication was the second most common reason for intentional medication non-adherence at 30.5%. The relatively low average age of participants suggests that this group may feel comfortable obtaining a prescription of medical cannabis even where wider societal stigma may remain, thus suggesting that medication adherence in EUPD patients may be improved with the use of CBMPs. A question remains: what is the end goal of a therapeutical approach using medicinal cannabis? Much like other medications used in EUPD, the aim is to achieve rapid symptom relief, allowing the patient to assimilate psychological techniques to manage their condition in the long term. In this way, CBMPs are not intended to supplant important psychological therapies needed for the management of EUPD but, rather, to act as an initial primer towards achieving symptom control and sparking patients with the hope of clinical improvement. 

Although promising, the results of this study are also hindered by the intrinsic limitations of the case series design. The small number of patients makes these early results highly preliminary and further research should be undertaken before robust conclusions about the efficacy of CBMPs for the management of EUPD can be drawn. Also, additional tools and questionnaires should be implemented to measure more granular nuances on the short-, mid-, and long-term psychiatric improvement of these patients associated with the treatment with CBMPs, as well as the potential deleterious effect of long-term administration of cannabinoids, especially THC. Finally, all patients reporting a benefit from the treatment with chemotype 1 *Cannabis flos* were already experienced cannabis users who were not required to discontinue their consumption prior to enrolling in the clinics for CBMPs treatment. This could potentially constitute a confounding factor, as naïve patients might have a different outcome. 

## 4. Conclusions

To our knowledge, this case series represents the first medical evidence of the use of CBMPs for the clinical management of patients with a diagnosis of EUPD, who are met with limited pharmacological options typically based on the off-label use of psychiatric medications. Cannabinoids may represent a novel, efficacious, and safe treatment alternative for EUPD patients. The neuro- and immune-modulatory effects of THC and CBD seem theoretically well-aligned with cellular and molecular deficits that are currently being investigated as key features underlying the pathogenesis of EUPD. Although preliminary, our results suggest that, when deployed in a rigorously controlled clinical environment, CBMPs can provide substantial improvement in symptoms associated with EUPD thus warranting the need for further research on this therapeutical strategy.

## Figures and Tables

**Table 1:** Basic demographics, clinical presentation, and substance use of seven patients presenting to Zerenia clinics with a diagnosis of EUPD. Current illicit cannabis users reported consuming every day or every other day. Occasional alcohol use represents less than 4 units a week.

#	Sex	Age	Occupation	Psychiatric Diagnosis	Comorbidities (List)	Past/Present Substance Use
1	F	24	Housekeeper	EUPD	Ehlers-Danlos syndrome	Occasional alcohol Non-smoker Current illicit cannabis use
2	M	24	Global Immigration Assistant	EUPD Anxiety Depression OCD	Migraines	Occasional alcohol Non-smoker Previous illicit/medical cannabis use Previous BZD dependency
3	F	34	Unemployed	EUPD	HTN	No alcohol Non-smoker Current illicit cannabis use Previous opiate dependency
4	M	28	Unemployed	EUPD GAD	None	No alcohol Non-smoker Previous illicit/medical cannabis use
5	F	46	Unemployed	EUPD Depression Treatment-resistant OCD	HTN, T2DM	No alcohol Non-smoker No history of cannabis use
6	F	25	Phlebotomist	EUPD Depression Anxiety	None	No alcohol Non-smoker Current illicit cannabis use
7	F	29	College student	EUPD Bipolar disorder Insomnia	None	No alcohol Non-smoker No history of cannabis use

Abbreviations: #, participant number; EUPD, emotionally unstable personality disorder; OCD, obsessive compulsive disorder; HTN, hypertension; T2DM, Type-2 diabetes mellitus; BDZ, benzodiazepine.

**Table 2:** Past and present treatment employed for the management of a wide range of symptoms seen in EUPD and other associated conditions.

#	Current Psychotropic Medication	Past Psychotropic Medication History	Talking Therapies Trialed
1	Citalopram 30 mg O.D.		DBT
2	None prescribed	Mirtazapine Duloxetine Sertraline Lamotrigine Quetiapine Sodium valproate Zopiclone	DBT
3	None prescribed	Sulpride Quetiapine Melatonin	CBT
4	Mirtazapine 30 mg O.N. Quetiapine 100 mg O.N.	Zopiclone Diazepam Propranolol	CBT Counselling
5	Duloxetine 90 mg O.D. Pregabalin 200 mg T.D.S Olanzapine 5 mg O.N.	Lorazepam Clomipramine Sertraline Fluoxetine	CBT
6	Fluoxetine 60 mg O.D.	Sertraline Escitalopram Citalopram	CBT Counselling
7	Paroxetine 12.5 mg O.D. Trazodone 50 mg O.D.	Lamotrigine	CBT

Abbreviations: #, participant number; O.D., omni die (once a day); O.N., omni nocte (once a night); T.D.S., ter die sumendus (three times a day); DBT, dialectical behavioural therapy; CBT, cognitive behavioural therapy.

**Table 3:** Cannabis-based medicinal product (CBMP), selected for each participant, with recorded CGI-I and PGIC scores at one-month follow-up. The differing CBMP preparations are shown with participants receiving either a dried flower (for vaping administration) or an oral extract. The preparations differ in their relative concentrations of THC and CBD as determined by the chemotype: chemotype 1 (THC >>> CBD), chemotype 2 (THC ≈ CBD), chemotype 3 (THC <<< CBD). For the flower preparations the dose is given in grams per month, as patients are often allowed to personalise their daily dose regime (typically 0.5–2 g a day) without exceeding the monthly dose, whereas the daily dose is recorded for extracts. CGI-I is recorded on a scale from 0 to 7 (0 = not assessed, 1 = very much improved, 7 = very much worse). PGIC is recorded on a scale from 1 to 7 (1 = no change or worse condition, 7 = a great deal better and a considerable improvement that has made all the difference.).

#	Cannabis Naïve	Cannabis-Based Medicinal Product Used	Adverse Effects	CGI-I	PGIC
1	No	Chemotype 1 dried flower 15–20% THC; 30 g per month	No side effects reported	2	6
2	No	Chemotype 2 Oral extract 10 mg/mL THC: 15 mg/mL CBD 0.5 mL B.D. Chemotype 1 dried flower 20% THC; 20 g per month	No side effects reported	2	5
3	No	Chemotype 1 dried flower 20% THC; 30 g per month	No side effects reported	2	6
4	No	Chemotype 1 dried flower 15–20% THC; 60 g per month	No side effects reported	2	6
5	Yes	Chemotype 2 Oral extract 10 mg/mL THC:12.5 mg/mL CBD 0.3 mL T.D.S.	No side effects reported	4	1
6	No	Chemotype 1 dried flower 20% THC; 30 g per month	No side effects reported	2	6
7	Yes	Chemotype 3 Oral extract 100 mg/mL CBD 0.4 mL O.D.	No side effects reported	2	6

Abbreviations: #, participant number; THC, tetrahydrocannabinol; CBD, cannabidiol; O.D., omni die (once a day); B.D., bis die (twice a day); T.D.S., ter die sumendus (three times a day); CGI-I, Clinical Global Impression–Global Improvement (CGI–I) Scale; PGIC, Patients’ Global Impression of Change (PGIC) scale.

## Data Availability

The data presented in this work is available on request from the corresponding author. The data are not publicly available due to privacy and ethical reasons.
